# Supplementary material for: Empirical methods for controlling false positives and estimating confidence in ChIP-Seq peaks
Source: BMC Bioinformatics. 2008 Dec 5;9:523. doi: 10.1186/1471-2105-9-523 (PMC2628906; doi:10.1186/1471-2105-9-523)
Supplement: Additional File 1 — A variety of html documents from the USeq web site detailing the available applications, their best usage, output file type descriptions, command line menus, etc. [file 1471-2105-9-523-S1.zip › USeqUserGuides/JavaDoc.zip!/JavaDoc/trans/main/class-use/Window.html]

Uses of Class trans.main.Window


|  |  |  |
| --- | --- | --- |
| **Overview** \| **Package** \| **Class** \| **Use** \| **Tree** \| **Deprecated** \| **Index** \| **Help** | |  |
| PREV   NEXT | **FRAMES**    **NO FRAMES**     **All Classes** |  |


---


## **Uses of Class trans.main.Window**

| Packages that use Window | |
| --- | --- |
| **trans.anno** | For associating intervals, binding regions, and binding peaks with GFF annotation. |
| **trans.main** | Principle classes and scripts used in TiMAT2 analysis. |
| **trans.misc** | Rough working scripts. |

Uses of Window in trans.anno

| Methods in trans.anno with parameters of type Window | |
| --- | --- |
| `boolean` | `Region.intersect(Window window, int sizeOligo, double fractionAcceptibleCoverage)`             Returns true only if region contains or is contained or is covered by >= fractionAcceptibleCoverage by a window. |

Uses of Window in trans.main

| Methods in trans.main that return Window | |
| --- | --- |
| `Window` | `MultiWindowIntervalMaker.checkWindowScoresAndNumberOfOligos()`             Checks the scores and number of oligos in each window. |
| `Window` | `MultiWindowIntervalMaker.fetchGoodWindow()` |
| `Window` | `IntervalMaker.fetchGoodWindow()` |
| `Window` | `SetNumberIntervalMaker.fetchGoodWindow(double minScoreInt)` |
| `Window[]` | `MergeWindowArrays.filter(Window[] win)` |
| `Window[]` | `WindowBlockMakerTwoColor.filterWindows(Window[] windows)`             Tosses any windows within the min max filter range. |
| `Window` | `MultiWindowIntervalMaker.findWindow(Window[] win)` |
| `Window` | `Interval.getBestWindow()` |
| `Window[]` | `ScanChromosomesCNV.getWindows()` |
| `Window[]` | `ScanChromosomes.getWindows()` |

| Methods in trans.main with parameters of type Window | |
| --- | --- |
| `boolean` | `WindowBlockMaker.baseOverlapsWindow(int bp, Window win)`             Is a given base contained within the window? |
| `boolean` | `IntervalMaker.checkScoreAndNumOligos(Window win)` |
| `Window[]` | `MergeWindowArrays.filter(Window[] win)` |
| `Window[]` | `WindowBlockMakerTwoColor.filterWindows(Window[] windows)`             Tosses any windows within the min max filter range. |
| `Window` | `MultiWindowIntervalMaker.findWindow(Window[] win)` |
| `void` | `ScanChromosomesCNV.importScores(Window[] windows, java.io.File importFile)`             Reads in a file of scores from a qvalue output file Also makes gr files for the qValues. |
| `void` | `ScanChromosomes.importScores(Window[] windows, java.io.File importFile)`             Reads in a file of scores from a qvalue output file Also makes gr files for the qValues. |
| `void` | `ScanChip.importScores(Window[] windows, java.io.File importFile)`             Reads in a file of scores from a qvalue output file Also writes and zips an .sgr file for the qValues. |
| `static void` | `IntervalMaker.invertScores(Window[] windows)`             Multiplies all of the Window[] scores by -1 |
| `void` | `ScanChromosomesCNV.makeBarFiles(Window[] windows, java.io.File directory, int scoreIndex, boolean stairStep)`             Makes xxx.bar.zip files from a Window[], one per chromosome. |
| `void` | `ScanChromosomes.makeBarFiles(Window[] windows, java.io.File directory, int scoreIndex, boolean stairStep)`             Makes xxx.bar.zip files from a Window[], one per chromosome. |
| `void` | `WindowBlockMakerTwoColor.makeHeatMapBarFiles(Window[] windows)`             Makes a stair step heat map from an array of windows in bar format. |
| `void` | `WindowBlockMaker.makeHeatMapBarFiles(Window[] windows, java.io.File directory)`             Be sure to set the scoreIndex, defaults to 0. |
| `void` | `WindowBlockMakerTwoColor.makeHeatMapBarFiles(Window[] windows, java.io.File barDirectory, int scoreIndex)` |
| `void` | `WindowBlockMaker.makeHeatMapGrFiles(Window[] windows, java.io.File directory)`             Be sure to set the scoreIndex, defaults to 0. |
| `void` | `WindowBlockMaker.makeHeatMapSgrFile(Window[] windows, java.io.File file, boolean zipCompressIt)`             Be sure to set the scoreIndex, defaults to 0. |
| `void` | `ScanChip.makeSgrFile(Window[] windows, java.io.File sgrFile, int scoreIndex)`             Makes a xxx.sgr.zip file from a sorted Window[]. |
| `boolean` | `Window.overlap(Window other)` |
| `boolean` | `WindowBlockMakerTwoColor.overlapOrAbut(Window left, Window right)`             Assumes right window is to right of left or abuts or doesn't overlap. |
| `void` | `Interval.setBestWindow(Window bestWindow)` |
| `void` | `FDRWindowConverter.setScoreAndSort(Window[] w)` |
| `int` | `FDRWindowConverter.thresholdWindows(Window[] w, double score, int startingIndex)` |

Constructors in trans.main with parameters of type Window

`Interval(Window window,
int sizeOfOligo)`

`IntervalMaker(java.io.File windowFile,
Window[] windows,
double[] cutoffs,
int sizeOfOligo,
int scoreIndex,
int maxGap,
int minimumNumberOfOligos)`

Uses of Window in trans.misc

| Methods in trans.misc that return Window | |
| --- | --- |
| `static Window[]` | `BestWindowScoreExtractor.makeWindows(java.io.File txtFile)`             Converts a tab delimited txt file of chr, start, stop, scores (multiple permitted or none) into an array of Windows. |
| `static Window[]` | `Util.removeLowOligoWindows(Window[] win, int minNumberOligos)`             Removes windows that contain < minNumberOligos. |
| `static Window[][]` | `Util.splitWindowsByChromosome(Window[] win)` |

| Methods in trans.misc with parameters of type Window | |
| --- | --- |
| `trans.misc.BestWindowScoreExtractor.QPCRWindow[]` | `BestWindowScoreExtractor.makeQPCRWindows(Window[] ws)` |
| `static double[]` | `Util.minMaxWindowArray(Window[] win, int scoreIndex)`             Returns the min and max values of a given score index in a Window[]. |
| `static double[]` | `Util.minMaxWindowScores(Window[] win, int scoreIndex)`             Finds min max score |
| `static Window[]` | `Util.removeLowOligoWindows(Window[] win, int minNumberOligos)`             Removes windows that contain < minNumberOligos. |
| `static Window[][]` | `Util.splitWindowsByChromosome(Window[] win)` |
| `static double` | `BestWindowScoreExtractor.windowMidPoint(Window w)` |

---


|  |  |  |
| --- | --- | --- |
| **Overview** \| **Package** \| **Class** \| **Use** \| **Tree** \| **Deprecated** \| **Index** \| **Help** | |  |
| PREV   NEXT | **FRAMES**    **NO FRAMES**     **All Classes** |  |


---
